# Peripheral Ulcerative Keratitis: A Review

**DOI:** 10.18502/jovr.v17i2.10797

**Published:** 2022-04-29

**Authors:** Kiana Hassanpour, Reem H. ElSheikh, Amir Arabi, Charles R. Frank, Abdelrahman M. Elhusseiny, Taher K. Eleiwa, Shiva Arami, Ali R. Djalilian, Ahmad Kheirkhah

**Affiliations:** ^1^Ophthalmic Research Center, Research Institute for Ophthalmology and Vision Science, Shahid Beheshti University of Medical Sciences, Tehran, Iran; ^2^Department of Ophthalmology, Faculty of Medicine, Cairo University, Cairo, Egypt; ^3^Department of Ophthalmology and Visual Sciences, University of Illinois at Chicago, Chicago, IL, USA; ^4^Department of Ophthalmology, Harvey and Bernice Jones Eye Institute, University of Arkansas for Medical Sciences, Little Rock, AR, USA; ^5^Department of Ophthalmology, Faculty of Medicine, Benha University, Benha, Egypt; ^6^Department of Medicine, Division of Rheumatology, University of Illinois at Chicago, Chicago, IL, USA; ^7^Department of Ophthalmology, Long School of Medicine, University of Texas Health at San Antonio, San Antonio, TX, USA

**Keywords:** Autoimmune Disease, Immunomodulatory Therapy, Ulcerative Keratitis

## Abstract

Peripheral ulcerative keratitis (PUK) is a rare but serious ocular condition that is an important clinical entity due to its ophthalmological and systemic implications. It is characterized by progressive peripheral corneal stromal thinning with an associated epithelial defect and can be associated with an underlying local or systemic pro-inflammatory condition, or present in an idiopathic form (Mooren ulcer). Associated conditions include autoimmune diseases, systemic and ocular infections, dermatologic diseases, and ocular surgery. Cell-mediated and auto-antibody-mediated immune responses have been implicated in the pathogenesis of PUK, destroying peripheral corneal tissue via matrix metalloproteinases. Clinically, patients with PUK present with painful vision loss, a peripheral corneal ulcer, and often adjacent scleritis, episcleritis, iritis, or conjunctivitis. Diagnostic evaluation should be focused on identifying the underlying etiology and ruling out conditions that may mimic PUK, including marginal keratitis and Terrien marginal degeneration. Treatment should be focused on reducing local disease burden with topical lubrication, while simultaneously addressing the underlying cause with antimicrobials or anti-inflammatory when appropriate. Existing and emerging biologic immunomodulatory therapies have proven useful in PUK due to autoimmune conditions. Surgical treatment is generally reserved for cases of severe thinning or corneal perforation.

##  INTRODUCTION

Peripheral ulcerative keratitis (PUK) is an acute destructive peripheral keratitis, associated with epithelial defect and stromal thinning in all cases. PUK is a sight-threatening condition that also carries a high risk of systemic morbidity and mortality when accompanied by an underlying collagen vascular disease, which occurs in almost half of noninfectious peripheral keratitis.^[1]^ PUK has an average incidence of 0.2–3 individuals per million population.^[2]^ Reviewing the current literature, the exact relationship of PUK among other inflammatory lesions of the peripheral cornea is ill-defined.

The current review discusses PUK as an immune-mediated keratopathy associated with systemic immune alterations. These systemic immune disturbances may be caused by an auto-immune disease, a systemic infectious condition, or a systemic condition neither autoimmune nor infectious. We will review the etiology, clinical aspects, pathophysiology, and treatment options of PUK, focusing on the updates of PUK pathogenesis and therapeutic advances.

### Features of the Peripheral Cornea

Compared to the central cornea, the corneal periphery differs in a few key ways. It contains characteristic features as a junction of cornea, conjunctiva, episcleral, and sclera.^[3]^ Histologically, the peripheral stroma has looser arrangements of collagen bundles, with the limbal vascular arcades and lymphatics extending about 0.5 mm into the clear cornea.^[4]^ Consequently, the corneal periphery has a higher density of Langerhans cells, immunoglobulin M, and C1 complement factor.^[3,5,6]^ Accordingly, any ocular or systemic condition that alters the dynamics of ocular surface immunity may be associated with an inflammatory response in the peripheral cornea. The peripheral corneal epithelium is more adherent to the underlying basement membrane with increased expression of cell surface-associated glycoprotein Mucin-4 gene (*MUC4*) that protects the corneal epithelium and controls the epithelial regeneration.^[4,7]^ In addition, the corneal periphery has proximity to the limbal epithelial stem cell niche and is the site with the maximum replicative activity of the corneal endothelium.^[4,8]^ Lastly, the corneal periphery is less sensitive owing to less neural innervation compared to the central regions.^[8]^

### Etiology of PUK 

**Figure 1:**
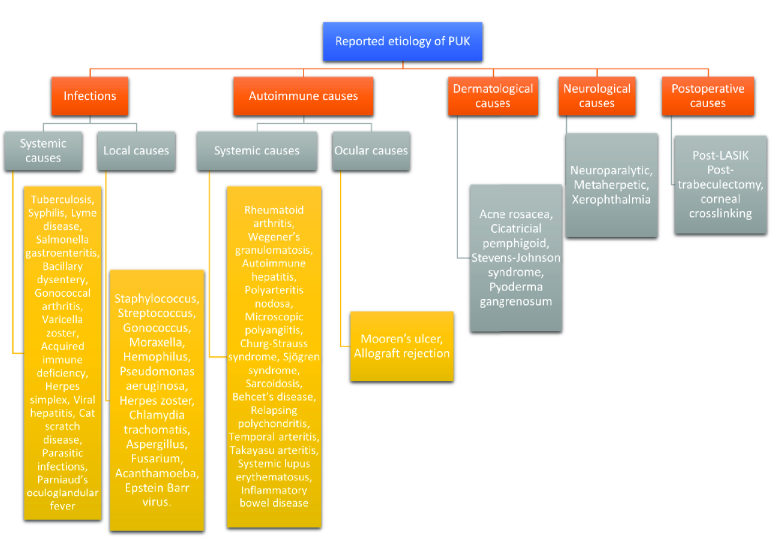
Various etiologies associated with peripheral ulcerative keratitis (PUK).

Peripheral ulcerative keratitis (PUK) can be caused by a variety of disorders including infectious and noninfectious conditions. Noninfectious causes include autoimmune, dermatological, neurological, and postsurgical etiologies.^[9]^ Diagnosis of the underlying cause is crucial to reducing mortality and systemic and ocular morbidities.^[10]^ Figure 1 lays out a classification schema for the etiology of PUK. Autoimmune diseases represent almost half of the noninfectious causes of PUK. Rheumatoid arthritis (RA) has been reported to account for 34% of noninfectious PUK with bilateral involvement in 50% of cases.^[1]^ Granulomatosis with polyangiitis (GPA) is a systemic vasculitis that is commonly associated with PUK in later stages.^[11]^ Other autoimmune etiologies of PUK include systemic lupus erythematosus (SLE), polyarteritis nodosa (PAN), progressive systemic sclerosis (PSS), Sjögren's syndrome (SS), relapsing polychondritis (RP), and giant cell arteritis (GCA).^[12]^ Dermatological causes include acne rosacea, which has been reported to have corneal involvement in 33% of patients.^[13]^

Infections, which have been reported as the second most common cause of PUK, are responsible for approximately 20% of all cases.^[14]^ Peripheral corneal infections must be associated with crescentic stromal thinning with ulceration and stromal inflammatory infiltrates to be defined as PUK.^[15]^ Maintaining a high index of suspicion for infectious etiologies of PUK is prudent, and they can be excluded by ordering relevant microbiological investigations based on the local prevalence of culprit organisms. Sharma and colleagues reported that 19.7% of infectious PUK was attributable to ocular sources, with bacteria representing 73.3% of cases.^[14]^ Systemic infections associated with PUK include bacterial (tuberculosis, syphilis, Lyme), viral (varicella-zoster), fungal, and parasitic etiologies.^[12]^

Sainz de la Maza and colleagues reported PUK in 7.4% of patients with scleritis.^[15]^ Also, they reported a high probability of PUK occurrence in the presence of necrotizing scleritis. In their small case series, all cases of scleritis-associated PUK had an underlying systemic vasculitis, which supports the hypothesis of coincidence of PUK and scleritis secondary to an extension of vasculitis from the sclera to peripheral cornea.^[15]^ Fluorescein angiography studies of the anterior segment have shown that PUK in scleritis is associated with vaso-occlusive changes of conjunctival and episcleral vasculature, where the larger the area of vascular occlusion, the greater the degree of corneal stromal lysis.^[16,17]^

PUK can present initial or be reactivated after ocular surgery. The site of disease is not necessarily close to the incision site. Severe cases of postoperative PUK have been reported in patients with associated Sjogren syndrome.^[18]^ Interestingly, despite RA being one of the most common causes of PUK, Jones and Maguire reported zero incidences of PUK during the eight-week follow-up period after cataract surgery in a cohort of 70 patients with RA.^[19]^ Retrospectively, Chanbour and colleagues reported late-onset PUK three to six months after corneal crosslinking in 1.4% of 771 eyes.^[20]^

As mentioned above, there is a broad range of potential causes of PUK. A systematic workup based on current knowledge and clinical presentation is paramount to refining the probabilities and guides for ordering the appropriate investigations and mapping the therapeutic options.

### Pathogenesis of PUK

Fundamental knowledge of the unique anatomy and physiology of the peripheral cornea is pivotal to understanding the pathogenesis of PUK. Subconjunctival lymphatics accompany the limbal capillaries into the peripheral cornea allowing access to the afferent limb of the immune system.^[21]^ An increase in inflammation via this vascular and lymphatic supply results in peripheral corneal ulceration due to the release of proteolytic enzymes from recruited cells.^[21]^ The limbal vascular architecture, along with tight corneal collagen packing at the periphery, facilitate deposition of immune complexes and other large molecules such as IgM and C1 at the corneal periphery.^[22,23]^ Antigen-presenting cells—Langerhan cells and limbal macrophages—present specifically at the limbus and peripheral cornea and act to propagate the immune-mediated corneal injury.^[24]^ In addition, the presence of activated T-cells in the tears of patients with underlying immune conditions may develop or exacerbate the immune reactions in PUK. Finally, in the humoral-mediated immune arm, auto-antibodies against corneal stromal antigens have been postulated to be involved in the pathogenesis of corneal destruction in PUK.^[21,25]^ Although many uncertainties exist regarding the exact pathogenesis of PUK, these common immune mechanisms have been implicated among the various types of PUK.

### PUK and T-cell Immunity

The polarization of the T-cell response and subsequent cytokine release plays an important role in protection against pathogens, as well as an important role in immunopathological conditions.^[26]^ The role of T-cell subsets in autoimmune disease has been widely discussed in the literature. Giscombe and colleagues concluded that the expansion of specific subsets of the T-cell population in correlation with disease activity suggests their role in the pathogenesis of that disease.^[27]^ For example, the number of CD4 cells was found to be significantly greater in patients with RA.^[28]^ Furthermore, the relative stability of these cell populations correlates with the chronic nature of a disease, suggesting a prolonged inciting stimulus.^[29,30]^ T-cells can be pathogenic through directly causing tissue damage or through unregulated auto-antibody and proinflammatory cytokine production.^[26]^

There is overwhelming evidence that adaptive T-cell mediated immunity is involved in the autoimmune mechanism in PUK. In a study conducted by Mondino and colleagues, seven patients with the clinical diagnosis of Mooren ulcer underwent macrophage migration inhibition factor testing to elucidate cellular immunity to corneal antigens. Six of these patients showed positive results suggesting that cell-mediated immunity may play a role in the pathogenesis of Mooren ulcer.^[31]^ In another study by Foster and colleagues on a patient with bilateral Mooren ulcers, an immune panel was normal except for a positive blastogenic response of the patient's T lymphocytes when exposed to the normal corneal stroma.^[32]^

Zhao and colleagues studied the immunological process involved in the development of Mooren ulcer and found a regulatory imbalance in a subset of the T-cell population, with a decline in the number of suppressor CD8 T-cells compared to CD4 T helper cells, while also finding that the peripheral cornea and surrounding conjunctival lymphatics are directly implicated in the immunopathogenesis of the disease process.^[33]^ Th17 cells expressing IL-17 along with other cytokines such as IL-1, IL-6, IL-17, IL-23 play a critical role in the pathogenesis of PUK in patients with GPA.^[34,35]^

### Role of Complement and Innate Immunity in PUK

The complement pathway can be activated via the classical pathway by antigen–antibody complexes, or via the alternative pathway by the direct binding of C3b.^[23,24]^ C1, which is the first component of the classical complement pathway, is activated by antigen–antibody complexes thus initiating the complement cascade. C1 is a large molecule, and its size hinders its uniform diffusion throughout the cornea, thus it resides mainly at the peripheral cornea and the limbus.^[36]^ Similarly, IgM is a large molecule (almost 900,000 times the size of IgA) causing it to be present in higher concentrations at the corneal periphery. Upon initiation of complement activation, a series of events ensues including the formation of small polypeptides such as C3a and C5a, which possess chemotactic activity. In contrast to C5a, which has well established chemotactic effects on neutrophils and eosinophils, it was only recently established that C3a causes selective activation of eosinophils which subsequently stimulate neutrophils through IL-8.^[37,38]^ Finally the complement system converges on the formation of the C5,6,7,8,9 complex, which is responsible for causing stromal destruction and lysis of cell membranes. This process has been implicated in peripheral corneal damage from PUK in RA as well as ANCA-associated vasculitis and has been considered a potential therapeutic target.^[39]^

Pathological examination of the corneas of patients with PUK revealed numerous proinflammatory cells of the innate immune system including neutrophils, mast cells, and eosinophils.^[9]^ It has been proposed that these cells are the source of destructive and collagenolytic enzymes that cause the subsequent tissue injury and corneal ulceration.^[40]^

### Role of B Cells and Antibodies in PUK

IgM is present with higher concentrations in the periphery of the cornea owing to its larger size and inability to diffuse centrally. Patients with RA have a loss of normal B cell tolerance to their antigens and some have serum IgM that is directed against their IgG (rheumatoid factor). These immune complexes can deposit in the peripheral cornea result in subsequent complement activation leading to corneal inflammation and ulceration as detailed above.^[21]^ Furthermore, anti-citrullinated protein antibodies (anti-CCP antibodies), also found in patients with RA, have been associated with more severe ocular findings.^[41]^

Patients with SLE have a disruption in the normally present B cell tolerance leading to the production of autoantibodies including antinuclear antibodies (ANA). These antibodies form immunocomplexes that affect the clearing of apoptotic cells. This starts a vicious cycle as the uncleared apoptotic cells result in more nuclear antigens that bind to ANA and cause more intense tissue damage. The damage extends also to areas devoid of immune complexes, as their presence in other areas and activation of complement results in the release of C3a and C5a, which perpetuate the inflammatory cycle and can lead to corneal melting.^[42]^ A similar process may occur in patients with GPA, where antineutrophil cytoplasmic antibodies (ANCA) bind to receptors on monocytes- and neutrophil—forming complexes that release proinflammatory enzymes and destructive cytokines such as IL-8.^[35]^

Antibodies directed toward corneal epithelium have been found in PUK patients who had GPA and RA. John and colleagues studied antibodies directed toward the cornea in GPA and RA patients who had PUK.^[11]^ They reported two corneal antigens of interest, with sizes of 54 kDa and 70 kDa. They showed that antibodies to the 54 kDa antigen, which is the main corneal specific antigen, appeared first after an attack of PUK, thus linking them to the immunopathogenesis of the disease.^[11]^ Reynolds and colleagues also studied two 66kDa corneal antigens, namely BCEA-A and BCEA-B, and the autoantibodies directed toward them. GPA and RA patients showed an increased level of BCEA-A where elevated levels of BCEA-B were found in patients with EGPA.^[43]^ Albers and colleagues reported the presence of circulating antibodies directed toward cornea in patients with RP.^[44]^

Mondino and colleagues reported that patients with Mooren ulcers had a consistent finding of tissue-fixed and circulating antibodies to conjunctival epithelium [PMID: 3050690]. Antibodies were found bound to the conjunctival basement membrane adjacent to the ulcerative lesion. Patients with Mooren Ulcer also demonstrated an elevated level of IgA.^[45]^ In another study by Gottsch and colleagues to study the level of antibodies toward cornea-associated antigen (CO-Ag) in patients with Mooren ulcer, they reported a statistically significant difference in the level of antibodies in the serum of patients with Mooren ulcer when compared to controls.^[46]^ It should be noted that the presence of these antibodies in the PUK patients does not prove causation, as the antibodies might be formed in response to the tissue damage incurred during the disease process.^[11]^

In addition to the production of autoantibodies, B cells in patients with RA are a source of cytokines that cause the pathological T-cell response.^[41]^ Specifically, TNF and IL-6 regulate downstream inflammatory cascades, and the latter is important in the regulation of the balance between T regulatory cells and Th17.^[47]^ In general, B cells are responsible for the regulation of the overall Th1/Th2 balance.^[48]^

### Role of Matrix Metalloproteinases in PUK

The corneal stroma is formed of organized collagen lamellae embedded in a frame of glycosaminoglycans. In between the adjacent lamellae are macrophages, lymphocytes, polymorphonuclear leukocytes, and fibroblasts known as keratocytes.^[49]^ MMPs are a family of endopeptidases, proteolytic enzymes that result in the degradation and breakdown of specific extracellular matrix components.^[50]^ The activity of MMPs is generally governed by their respective tissue inhibitors (TIMP).^[51,52]^ Imbalance and reduced expression of TIMP result in high collagenase activity and increased tissue destruction and ulceration. Although different subsets of MMPs exhibit substrate specificity, they have an overall similar mechanism of action and structure. MMPs can be classified according to substrate specificity and structural organization into collagenases (MMP-1, -8, and -13), gelatinases (MMP-2, -9), stromelysins (MMP-3, -10), matrilysins (MMP-7, -26), membrane-type MMPs (MT1eMT6-MMPs), and others (e.g., macrophage metalloelastase MMP-12 or enamelysin MMP-20).^[53]^

MMP-1, produced by macrophages and fibroblasts, and MMP-8, produced by neutrophils and invading inflammatory cells near the limbus, have an established pathogenic role in the etiology of PUK.^[54]^ They are the only known mammalian enzymes known to be able to initiate hydrolysis of fibrillar type 1 collagen, the main component of the corneal stroma.^[54]^ The gelatinases (MMP-2, MMP-9) can cleave basement membrane components (collagen types IV and VII, fibronectin, and laminin) and stromal collagen types IV, V, and VI, the core protein decorin and denatured collagens.^[54,55]^

Smith and colleagues reported upregulation in the level of MMP-2 in keratocytes derived from corneas of patients with PUK and accumulation of MMP-9 in tears of patients with active disease.^[56]^ Although most theories linked the collagenases, namely MMP-1, and/or the reduction of their TIMP to the pathogenesis of PUK, Smith and colleagues suggested that activated gelatinases are also linked to the progression of PUK. They may cause corneal perforation by breaching the corneal basement membranes (epithelial cells and Descemet membrane).^[56]^ They could also impede the process of tissue repair leading to chronicity by breaking down the newly formed non-crosslinked collagen.

### PUK Associated with Systemic Noninfectious Diseases

The most frequent systemic noninfectious diseases associated with PUK are systemic collagen vascular diseases, accounting for nearly half of PUK cases.^[1]^ Around one-third of PUK cases is associated with RA.^[57]^ Following RA, ANCA-vasculitis is an autoimmune disease commonly associated with PUK. Other associated diseases include SLE, polyarteritis nodes (PAN) and its variants, Sjorgren's syndrome, RP, and eosinophilic granulomatosis with polyangiitis (EGPA) [Table 1]. ${}^{[}$ 36, 58–60 ${}^{]}$ PUK can be the presenting sign of autoimmune systemic diseases, or it can be manifested in a patient with previously diagnosed disease. Also, it can be the only ocular surface complication of the underlying systemic disease, or it may develop in association with necrotizing scleritis.^[21]^ Visual outcomes are poor in half of the PUK cases associated with systemic immune diseases, despite topical and systemic therapy.^[61]^

**Table 1:** Some systemic noninfectious diseases associated with PUK, and their key findings necessary for an ophthalmologist to know


**The systemic condition associated with PUK**	**Key demographic features**	**Key systemic findings suggesting the diagnosis**	**Suggestive diagnostic evaluations**
**Non-ANCA associated small-vessel vasculitis**			
Rheumatoid arthritis	Between 35 and 50 years, more common in women	Symmetric polyarthritis, morning stiffness, hand joints involvement, rheumatoid sub-cutaneous nodules	Presence of rheumatoid factor, typical radiologic findings
SLE	90% of patients are women, between 15 and 45 years	Fatigue, fever, joint pain, stiffness and swelling, butterfly-shaped rash on the face that covers the cheeks and bridge of the nose Skin lesions that appear or worsen with sun exposure	ANA positive, anti-ds DNA positive, anti-sm positive
Sjogren's syndrome	Between 40 and 60 years, more common in women	Dry eye and mouth	Anti-LA and anti-Ro positive
**ANCA-associated small-vessel vasculitis**			
Wegener's granulomatosis	Between 55 and 70 years, more common in men	Nasal sinus inflammation, abnormal urine analysis, pulmonary signs and symptoms	c-ANCA positive, granulomatous inflammation and necrotizing vasculitis on histopathology
Microscopic polyangiitis	All ages, more common in men	Fever, loss of appetite and weight, rashes, muscle and/or joint pain,, pulmonary signs and symptoms neuropathic signs and symptoms, gastrointestinal signs and symptoms	p-ANCA positive, patchy 3-layer inflammation of small arteries and vein on histopathology
Churg-Strauss syndrome	Mean age of onset 40 years, more common in female	Asthma, peripheral neuropathic signs, and symptoms, transient pulmonary infiltration	Prominent eosinophilia, extravascular eosinophils on histopathology
**Medium-sized vessel vasculitis**			
PAN	Between 40 and 50 years, more common in men	Fever, weight loss, palpable purpura, skin ulceration and infarction, recent-onset HTN, testicular pain	HBV serology, focal vascular stenosis, or microaneurysm on CTA/MRA
**Large-sized vessel vasculitis**			
Giant cell arteritis	Over 50 years, more common in women	Headache and scalp tenderness, jaw claudication	Elevated ESR and CRP
Takayasu's disease	Under 40 years, more common in men	Claudication of the extremities, the decreased pulse of brachial arteries, bruit over subclavian arteries	Arteriographic narrowing of the aorta
**Other immune diseases**			
Behcet disease	Between 20 and 50 years, equally prevalent among men and women	Ocular or genital aphthous	Positive pathergy test, positive HLA-B51
Sarcoidosis	Between 20 and 60 years, more common in women	Pulmonary signs and symptoms, lymphadenopathy, skin lesions	Elevated ACE and lysozyme
IBD	Between 15 and 30 years, equally prevalent among men and women	Gastrointestinal signs and symptoms	Abnormal stool studies, characteristic colonoscopy findings
Progressive systemic sclerosis	Between 40 and 50 years, more common in women	Raynaud phenomenon, sclerodactyly, skin telangiectasia	Clinical diagnosis
Relapsing polychondritis	Between 40 and 60 years, equally prevalent among men and women or more common in women	Fever, painful erythematous plaques, inflammation of nasal and auricular inflammation	Clinical diagnosis
**Other immune diseases**			
Psoriasis	Between 20 and 50 years, more common in women	Red skin patches with silver scales	Clinical diagnosis
Pyoderma gangrenosum	Between 15 and 30 years	Painful skin lesions start with a small, red bump on the skin	Clinical diagnosis
**Malignancies**			
Acute myelogenous leukemia	Over 50 years, more common in men	Fever, bone pain, lethargy and fatigue, pale skin	Abnormal WBC counts
Chronic myelogenous leukemia	Over 50 years, more common in men	Fever, bone pain, lethargy and fatigue, pale skin	Abnormal peripheral blood cell counts
	
	

**Table 2:** Common immunosuppressive agents with their complications


**Drug category**	**Drug name**	**Mechanism of action**	**Common complications**	**Alarming signs of complication occurrence**	**Monitoring of complications**
**Biologic agents**	Etanercept	TNF inhibitor	Opportunistic infections, lymphoma, injection site reactions, cutaneous vasculitis, multiple sclerosis, congestive heart failure	Mouth ulcers, fever, chills, bruising, pallor	Blood count and liver function test, 1–3 monthly dsDNA – yearly
	Infliximab	TNF inhibitor	Opportunistic infections, lymphoma, injection site reactions, cutaneous vasculitis, multiple sclerosis, congestive heart failure	Mouth ulcers, fever, chills, bruising, pallor	Blood count and liver function tests before each infusion dsDNA – yearly
	Adalimumab	TNF inhibitor	Opportunistic infections, lymphoma, drug-induced lupus, worsening or the initiation of congestive heart failure, cytopenias, worsening or the initiation of multiple sclerosis/neurological disease	Mouth ulcers, fever, chills, bruising, pallor	Blood count and liver function tests monthly for 3 months; then 3 monthly dsDNA – yearly
	Rituximab	Anti CD-20	Cardiovascular and dermatological diseases, cytopenia, opportunistic infections, infusion reaction	Fatigue, pallor, dyspnea, fever, chills, abdominal pain, pruritus	Vital sign monitoring at each ophthalmic visit, blood count regularly
	Daclizumab	IL-2 inhibitor	Granulomatous inflammation, opportunistic infections	Lymphadenopathy, rash, sore throat, pallor, fever, chills	Blood count regularly
**Alkylating agents**	Cyclophosphamide	Impaired cell proliferation due to DNA damage	Cytopenia, mucositis, cystitis, pneumonia, opportunistic infections	Fever, bruising, pallor	Blood count, liver function test, and urine exam 10 days after the last dose and 2 days before next
	Chlorambucil	Impaired cell proliferation due to DNA damage	Cytopenia, mucositis, cystitis, pneumonia, opportunistic infections	Fever, bruising, pallor	Blood count, liver function test, and urine exam 10 days after the last dose and 2 days before next
**T cell inhibitors**	Cyclosporine	Transcriptional suppression of T cells	Hypertension, cytopenia, nephrotoxicity	Peripheral edema, mouth ulcer, rash Increased blood pressure	Blood count every 2 days for 6 weeks; then 1–2 monthly Liver function tests monthly especially if concomitant NSAIDs Lipids every 6 months
	Tacrolimus	Transcriptional suppression of T cells	Hypertension, cytopenia, nephrotoxicity	Peripheral edema, mouth ulcer, rash Increased blood pressure	Blood count every 2 days for 6 weeks; then 1–2 monthly Liver function tests monthly especially if concomitant NSAIDs Lipids every 6 months
**Antimetabolites**	Methotrexate	Impaired DNA metabolism	Gastrointestinal signs and symptoms, cytopenia, increased liver enzymes	Fever, bruising, pallor, mouth ulcer, respiratory signs, and symptoms	Blood count and liver function test biweekly for 1 month; monthly for 6 months; then if stable, extend to 2–3 monthly
	Azathioprine	Impaired DNA metabolism	Gastrointestinal signs and symptoms, cytopenia, increased liver enzymes	Fever, bruising, pallor	Blood counts and liver function test monthly for 6–12 months; if stable extend to 6–8 weekly
	Mycophenolate mofetil	Impaired DNA metabolism	Gastrointestinal signs and symptoms, cytopenia, increased liver enzymes, respiratory symptoms, hematuria	Fever, bruising, pallor, mouth ulcer	Blood count weekly for 4 weeks; then monthly Liver function test and ESR and CRP monthly
	
	

**Table 3:** Differential diagnoses for PUK


**PUK differential diagnoses**	**Distinguishing clinical features**	**Management**
**Marginal keratitis and catarrhal infiltrates**	Dense white-gray stromal infiltration, the lucid interval between the infiltration and the limbus, more common in corneal sections in contact with the eyelid margins, associated with bacterial blepharitis detectable in the slit-lamp examination, mild to moderate ocular surface symptoms	Topical antibiotics with eyelid hygiene Corneal involvement rapidly responds to topical corticosteroids Any suspicion for bacterial keratitis should prompt immediate microbial smear and culture
**Rosacea-associated peripheral keratitis**	Circumferential superficial stromal infiltration with vascularization, prominent eyelid margin signs and MGD, facial pustular rashes may be present	Systemic treatment with antibiotics, especially tetracycline and doxycycline
**MGD-associated peripheral keratitis**	Superficial corneal infiltration and neovascularization, conjunctival injection associated with meibomitis and eyelid margin telangiectasia	Eyelid hygiene with topical antibiotics and anti-inflammatory agents
**Corneal phlyctenulosis**	Subepithelial inflammatory nodule initially evident on the limbus, no lucid interval	Topical corticosteroids Treatment of concomitant blepharitis Consider TB in endemic regions and child patients
**Peripheral infectious keratitis**	May be associated with hyper-acute conjunctivitis, history of corneal HSV infection, presence of underlying exposure keratitis, or history of contact lens wearing	Appropriate topical antibiotics or antiviral agents against Neisseria Gonorrhea, *Hemophilus* influenza type 2, Streptococcus, HSV, VZV, *Pseudomonas* Conservative measures for corneal repair Consider systemic antibiotics in Gonorrhea
**Peripheral corneal degenerations**	
Terrien's marginal degeneration	Progressive noninflammatory, peripheral corneal thinning, associated with corneal neovascularization, opacification, and lipid deposition New-onset against-the-rule astigmatism	Annular keratoplasty or lamellar graft in severe cases with prominent corneal thinning and progressive decreased vision
Furrow degeneration	Decreasing width of the peripheral cornea between the arcus senilis and limbus	No therapy is required
**Contact-lens induced peripheral infiltration**	Sudden-onset, small, circular, focal anterior stromal infiltrate in the corneal periphery or mid periphery, associated with a significant overlying epithelial loss, resolving to corneal scars	Contact lens removal Optional topical antibiotics Any suspicion for bacterial keratitis should prompt immediate microbial smear and culture
**Mooren's ulcer**	Peripheral corneal inflammation and thinning spreading circumferentially and centrally, lack of scleritis, lack of a systemic condition	Topical and systemic immunosuppressant
**Corneal Dellen**	Peripheral corneal thinning due to stromal dehydration, associated with a paralimbal elevation that can induce a localized break in the precorneal oily layer of the tears	Lubrication and patching A bandage contact lens may be required
**Exposure keratopathy**	Incomplete eyelid closure, more common following eyelid surgeries or admitted patients, persistent epithelial defects in the inferior or central cornea with or without dense stromal infiltration	Intensive lubrication and eyelid taping Consider transient or permanent tarsorrhaphy Any suspicion for bacterial keratitis should prompt immediate microbial smear and culture
	
	

Due to the diversity in the prevalence of different systemic autoimmune diseases, it is difficult to determine the exact epidemiology of PUK in these patients. According to one report, PUK was the second most common ocular complication of autoimmune diseases, following anterior uveitis.^[62]^ From a clinical and pathophysiological perspective, it is impossible to distinguish between PUK due to different systemic immune diseases, although some studies emphasize the function of CD4+ T-cells in PUK secondary to RA and auto-antibodies in PUK associated with SLE and GPA.^[63]^ Also, it has been postulated that PUK is more common in long-standing RA, while in GPA and another vasculitis, it can occur earlier in the course of the disease.^[63,64]^ However, PUK cases are mainly distinguished by differences in the clinical presentation, biomarkers, and other auxiliary examinations of the underlying systemic disease. Although traditionally it is believed that PUK occurs when the systemic disease is not controlled properly, in a recent series of PUK patients with underlying RA and Sjorgren's syndrome, > 80% of PUK episodes arose before autoimmune disease diagnosis or with the systemic disease in remission by immunosuppressive treatments.^[61]^ Any patient with PUK must have a detailed personal and family history elicited, and additional diagnostic tests should be considered in all cases with suspicious clinical signs or symptoms suggesting systemic collagen vascular diseases.

RA- and ANCA-associated systemic vasculitic diseases are not the only associates of corneal ulcerations. PUK is also an ocular complication of sarcoidosis and Behcet's disease. Sarcoidosis is a granulomatous inflammation associated with systemic vasculitis.^[27]^ With the involvement of the small vessels in the vasculitic course of sarcoidosis, it can be complicated by potential corneoscleral inflammation, even as the first manifestation of the systemic disease.^[65,66]^ Similarly, PUK can develop in patients with underlying Behcet's disease, although it is rare and poorly documented.^[67]^ Keratopathy is a rare manifestation of inflammatory bowel disease (e.g., Crohn's disease and ulcerative colitis), which is commonly associated with subepithelial infiltrates.^[68]^ However, there are reports regarding the development of PUK with corneal perforation in Crohn's disease.^[69]^

Among dermatologic immune diseases, PUK has been reported in parallel to an exacerbation of psoriasis.^[70]^ Inflammatory ocular complications of psoriasis may occur in only 5% of patients, and corneal involvement is even less common. However, psoriasis-associated keratopathy can be equally severe as other ocular conditions, threatening patients' vision.^[70]^ PUK has also been associated with severe hidradenitis suppurativa (HS).^[71,72]^ As a suppurative and cicatricial disease of the apocrine glands of the skin, HS is not generally considered as an autoimmune disease, and treatment is targeted to quiet the inflammatory stages of the disease, rather than treating a major underlying autoimmune process.^[72]^ However, underlying immune dysregulation in HS patients may be associated with the risk of severe ocular surface diseases.

Although systemic autoimmune diseases are the primary etiology of noninfectious PUK, systemic malignancies should also be considered as etiologic factors. There are reports of PUK associated with both acute and chronic myelogenous leukemia, emphasizing the importance of a careful diagnostic approach, especially in cases with no underlying immune disease. ${}^{[}$ 73–75 ${}^{]}$ Deposition of leukemic blast cells in the limbus and subsequent inflammatory reaction has been implicated in the pathophysiology of leukemia-associated PUK. This hypothesis was derived from the time of PUK incidence in acute and chronic leukemia. In patients with chronic myelogenous leukemia, most PUK cases have occurred in the blast crisis phase resembling acute leukemia. On the other hand, PUK was detected in an untreated acute leukemia patient, when malignant cells were present in the circulating system.^[75]^ Similar pathogenesis may be responsible for PUK in pyoderma gangrenosum, where an underlying immune dysregulation causes neutrophilic dermatoses characterized by the infiltration of neutrophils throughout the dermis. ${}^{[}$ 59, 76–79 ${}^{]}$ PUK associated with systemic malignancies and pyoderma gangrenosum shares similarities with immune disease-associated PUK.

### PUK Associated with Systemic Infectious Diseases

Apart from noninfectious disorders, some systemic infectious conditions have also been associated with PUK. Systemic TB can predispose both pediatric and adult patients to PUK.^[80]^ Immune reactions in TB patients can be manifested by uni- or bilateral corneal ulceration and interstitial keratitis, and it can occur in peripheral as well as central cornea.^[81,82]^ PUK is a rare complication of syphilis, while stromal keratitis is the characteristic corneal involvement of these patients. PUK associated with syphilis can be the presenting sign of acquired syphilis, and it can be complicated by corneal perforation. The pathogenesis most probably involves the immune reactions to spirochetal antigens.^[83,84]^ Bilateral PUK has also been reported following an episode of Bartonella infection in conjunction with bilateral conjunctivitis.^[85]^

The number of PUK cases as the presenting sign of human immunodeficiency virus (HIV) infection is growing through the literature.^[86,87,88,89]^ Human HIV-associated vasculopathy can result in immune complex-mediated reactions, and the limbus can be the primary site of the inflammation. Peripheral keratitis has also been reported following immune reconstruction associated with the initiation of HIV treatment.^[90]^ Both hepatitis B and C viruses have been associated with PUK, where hepatitis-associated cryoglobulinemia has been implicated in the pathogenesis. Cryoglobulinemia is more commonly observed with the hepatitis C virus and can cause vasculitis through deposition in small vessels. Additionally, hepatitis B infection has been associated with inflammation of medium-sized vessels, an entity previously known as HBV-related PAN. Accordingly, limbal vasculitis, whether through cryoglobulinemia or direct vessel inflammation, can cause PUK in hepatitis.^[91,92,93,94,95]^

### PUK Following Ocular Surgeries

PUK can occur after corneal surgeries, where corneal trauma induces immune reactions through altered corneal antigens. Both PUK and Mooren's ulcers have been reported to occur following ocular surgeries, including cataract surgery, laser in situ keratomileuses (LASIK), and penetrating keratoplasty (PK).^[96,97,98,99,100]^ It can happen weeks to months after uncomplicated cataract surgery through small corneal incisions.^[99,101]^ There is some controversy over whether postoperative PUK is truly a unique entity or whether it is simply a sign of underlying systemic disease. The occurrence of postoperative necrotizing scleritis and keratitis in patients with previously diagnosed systemic vasculitis is documented in the literature.^[100]^ For practical purposes, mild postsurgical keratitis in patients with no underlying disease may be considered to be isolated, but in severe cases, it is reasonable to perform a detailed systemic evaluation.

### Clinical Presentation of PUK

The main symptoms of PUK are photophobia, ocular redness, pain, and loss of vision secondary to either inflammation in the acute stage or scar-induced astigmatism in the chronic stage. On slit-lamp examination, the main signs include peripheral corneal crescentic stromal thinning, ulceration with overhanging edges, and mild to severe cellular infiltrates. Corneal perforation is a rare serious complication in PUK.^[102,103]^ Varying degrees of corneal neovascularization surround the crescent-shaped peripheral corneal lesion. Generally, PUK involves a single corneal section and is unilateral, but bilateral asymmetric cases have been also reported.^[57]^

PUK may be associated with iritis, conjunctivitis, episcleritis, or scleritis. The presence of scleritis can influence the final prognosis since it can aggravate the course of PUK and increase the rate of complication.^[64,104]^ The severity of corneal inflammation correlates with the concomitant scleritis severity, which is explained by the similar pathologic processes manifested by collagenolysis in both conditions.^[105]^

### Differential Diagnoses of PUK

Any peripheral corneal disease associated with stromal infiltration or thinning can be considered in the differential diagnosis of PUK [Table 2].

### Immune-mediated peripheral infiltration and ulceration associated with ocular surface diseases

Marginal keratitis represents an immune reaction to staphylococcal antigens. It can occur in patients with symptomatic blepharoconjunctivitis, or it can be associated with asymptomatic staphylococcal colonization of the eyelids.^[106]^ Also known as catarrhal infiltrates, marginal keratitis can occur secondary to the eyelid infection by other microorganisms, such as Hemophilus, Moraxella, or Streptococcus.^[107,108]^

Distribution of bacterial antigens through tear film and their presence in the peripheral cornea induces a type-III hypersensitivity reaction, where in-situ production and deposition of immune complexes trigger keratolysis and stromal infiltration.^[24]^ The presence of a clear area between the ulcer and the adjacent limbus has traditionally been used to distinguish marginal keratitis from other conditions such as PUK and phlyctenulosis. As another distinguishing feature, marginal keratitis responds rapidly to topical treatment, while PUK almost always requires appropriate systemic medication.

Phlyctenular corneal disease is another immune-mediated peripheral corneal lesion most commonly observed in association with longstanding staphylococcal blepharoconjunctivitis. Phlyctenules are subepithelial nodules that first appear in the limbus and extend toward the cornea later in the course of the disease. Rarely, corneal phlyctenules form a corneal ulcer. Historically, there was a strong association between tuberculosis and phlyctenulosis, but more recent studies show that staphylococcal disease is the most frequent etiology.^[24,57]^

Corneal involvement can occur in up to half of the patients with ocular rosacea, ranging from mild punctate epithelial keratitis to corneal ulceration and perforation. Rosacea-associated keratitis is characterized by a spade-shaped triangular vascular lesion associated with subepithelial and stromal infiltration. Occasionally, it can be complicated by significant peripheral corneal thinning, corneal melting, and perforation.^[109]^

Corneal infiltration and epithelial defects associated with meibomitis have been explained through the literature as meibomitis-related keratopathy or keratoconjunctivitis (MRKC).^[110,111]^ It is more common in young patients with severe meibomitis. MRKC may be manifested as a phlyctenule-like lesion with subepithelial infiltration, or it can be detected by superficial epithelial defects without stromal infiltration. The severity of MRKC has been correlated to the severity of MGD, the treatment of meibomitis is necessary for the remission of corneal complications.^[112]^

### Post-infection peripheral keratitis

Herpetic simplex virus (HSV) keratitis should always be considered as an important differential diagnosis of central or peripheral keratitis. As opposed to marginal keratitis, HSV infection of the cornea starts with epithelial defect and evolves to ulcer through subsequent subepithelial infiltration. Compared to other corneal ulcers, HSV-induced keratitis is less symptomatic, which is explained in part by the presence of decreased sensation in the infected corneal neurons. PUK-like lesions have also been reported in herpes zoster ophthalmicus associated with anterior uveitis.^[113]^

Neisseria gonorrhea is a virulent micro-organism that can penetrate the intact corneal epithelium. Chemotic conjunctiva during the hyper-acute phase of gonorrheal conjunctivitis overlaps the peripheral cornea, exposing it to a recess of bacteria and enzyme-laden material. Peripheral ulcers can evolve following primary stromal infiltration, and there is a prominent risk of corneal perforation.^[108]^ Similarly, *Hemophilus* influenza type 2 and streptococcal hyper-acute conjunctivitis may be associated with a peripheral corneal ulcer, characterized by a mucopurulent base and an overhanging edge.^[114]^ Peripheral corneal ulcers due to Moraxella occur following a defect in corneal epithelium, usually secondary to exposure keratitis in a debilitated patient, in the setting of a mild blepharoconjunctivitis (compared to the more severe conjunctivitis in gonorrhea or Streptococcus infections).^[115]^ Peripheral keratitis secondary to *Pseudomonas* is less common than central corneal involvement and commonly occurs in contact lens wearers and ill patients.^[116]^

###  Degenerative peripheral corneal diseases

Terrien's marginal degeneration is a slowly progressive noninflammatory corneal thinning. It can be uni- or bilateral, and is commonly associated with peripheral corneal opacification, neovascularization, and lipid deposition. Terrien's degeneration can be diagnosed following new-onset against-the-rule or oblique astigmatism. The peripheral thinning spreads circumferentially, starting from the superior limbus and rarely involving the inferior cornea. The corneal epithelium is usually intact over the thinning area, and the furrow may be crossed by superficial vessels. The condition can be complicated by corneal perforation following mild trauma, or it can develop acute corneal cysts due to Descemet's membrane ruptures.^[117,118,119]^

Another noninflammatory peripheral corneal thinning, furrow degeneration, is characterized by narrowing of the peripheral cornea between the arcus senilis and limbus. As a distinguishing feature, furrow degeneration lacks corneal vascularization, compared to Terrien's degeneration. Also, it is rarely associated with visual disturbance, and the risk of corneal perforation is extremely rare.^[120,121]^

Corneal Dellen is caused by stromal dehydration and subsequent excavation of the peripheral cornea associated with a paralimbal elevation. The pathogenesis is related to a break in tear film overlying the peripheral cornea beyond the elevated conjunctiva. If untreated, corneal dellen can be complicated by perforation, but use of artificial tears and patching, in association with treating the elevated paralimbal tissue, helps the cornea to heal within a few weeks.^[122,123,124]^

### Mooren Ulcer 

Mooren ulcer is the most similar entity to PUK, and can essentially be thought of as an idiopathic form of PUK. The pathophysiology of Mooren ulcer is outlined in detail below; it is thought to be an autoimmune condition triggered by cornea-specific antigens that become exposed due to trauma or surgery.^[125]^ It generally presents either as a chronic unilateral slowly progressive ulcer in older adults or bilateral rapidly progressing ulcers in younger adults. The pain is characteristically more severe and intolerable than PUK, with associated redness and photophobia. By definition, there is no underlying causal systemic inflammatory condition (as opposed to PUK), although Mooren has been associated with helminthic infections,^[126]^ exposure to viruses (hepatitis C), and the presence of HLA-DR17 or DQ2 antigens.^[127]^

Mooren ulcer starts at the limbus and proceeds to spread circumferentially and then centrally. It is most commonly found nasally or temporally adjacent to the interpalpebral limbus, without the clear area found in Terrien's (which is also typically painless).^[18]^ There is no scleral involvement, as opposed to PUK, which often has adjacent scleritis.^[18]^ There is usually associated thinning, and the bed of the ulcer can become vascularized. Eventually, it can progress to a complete circumferential defect, leaving only a central opaque and edematous intact cornea. In severe cases, the central cornea becomes consumed by a fibrovascular membrane as well, and perforation can occur.^[51]^

The therapeutic regimens for Mooren ulcer share many similarities with those for PUK, where the mainstay of the treatment is systemic immunosuppression.^[128,129]^ Topical treatment with corticosteroids, acetylcysteine, cyclosporine, and interferon-α ${}_{2a}$ have been used with limited effect. Surgical options include removal of the antigen source (lamellar keratoplasty), removal of the site of immunologic response (limbal/conjunctival resection), and tissue reinforcement to avoid perforation (patch grafts). There is no randomized control trial data to support any of the above treatment options, and no high-quality evidence supports the use of any one treatment.^[130]^

### Management

The first principle in approaching the treatment of PUK is to address the underlying cause if one is present. This is fundamental for reducing the ocular morbidity and potential mortality associated with systemic inflammatory conditions.^[9]^ Beyond this, the current management paradigm consists of treatment during the acute phase with systemic immunosuppression, usually a corticosteroid and immunomodulatory agent. This is followed by gradual tapering of the corticosteroid and maintaining the immunomodulatory agent to avoid disease recurrence. Throughout the disease course, aggressive topical immunosuppression and lubrication should be used to minimize stromal loss and optimize the ocular surface. If at any point there is concern for corneal perforation or worsening ulceration despite maximum medical therapy, early surgical intervention is indicated.^[12]^ Despite recent advances in immunosuppression therapeutic regimens, early diagnosis and optimal control of the accompanying systemic diseases remain the main prognostic factors in PUK.

### Topical Treatment

In cases of suspected infectious PUK, empirical topical antimicrobial therapy should be started after collecting corneal scrapings for microbiological examination. Knowing the local disease epidemiology, meticulous history taking, and systems review are key factors to reaching the causative organism. For instance, testing for TB in countries like India, China, Pakistan, Philippines, Bangladesh, and South Africa will have more likelihood of being positive because of the high prevalence of TB in these areas.^[12]^ For suspected bacterial keratitis, empirical monotherapy with a fourth-generation fluoroquinolone is usually started till being replaced with specific therapy according to the results of the culture. To suppress the coexisting immune-mediated inflammatory PUK, topical steroid therapy is usually started after 3 to 7 days of response to antibacterial treatment, or 14 days after starting the antifungal therapy in case of bacterial or fungal PUK, respectively. For PUK with suspected herpetic etiology, topical antiviral acyclovir or ganciclovir are prescribed.

In noninfectious PUK, local treatment is a cornerstone of symptom management and slowing the progression of corneal disease in PUK. Topical lubrication, which has the combined effect of improving the quality of the tear film and reducing discomfort, remains the mainstay treatment of PUK. In addition, frequent use of preservative-free artificial tears will help washout the inflammatory mediators, decrease immune-mediated keratolysis.^[12]^ Some of the available options include carboxymethylcellulose (0.25%, 0.5%, 1%), hydroxypropyl methylcellulose (0.3%), sodium hyaluronate (0.1%), polyethylene glycol (0.4%, 1%), and glycerine (1%). The frequency of instillation depends on the severity of keratoconjunctivitis sicca. Nighttime application of lubricant ointment formulations will promote epithelial healing as well.^[12]^

To more specifically target keratolysis, MMP inhibitors may be useful. MMPs include two groups of zinc-related peptidases: collagenases and gelatinases, which play a role in the degradation of the corneal stroma, as discussed above.^[131]^ N-acetylcysteine (NAC) chelates MMP-associated calcium or zinc, causing irreversible inhibition of MMPs, and has also been shown to reduce the release of various inflammatory cytokines in animal models.^[132]^ Despite the link between MMPs and PUK, and the clear *in vitro* inhibitory effects of NAC on MMPs, its clinical efficacy in the treatment of PUK is not well established.^[50]^

Corticosteroids are an obvious potential therapeutic option for PUK, given their potent anti-inflammatory properties. Selection of potency and frequency of instillation depends on the severity of the inflammation to be controlled. The potency of steroids may be low as in fluorometholone 0.1%, loteprednol etabonate 0.2% and 0.5%; moderate as in prednisolone sodium phosphate 0.5%, betamethasone 0.1%, dexamethasone 0.1%; or high as in prednisolone acetate 1%. Dosing is usually started four times a day, then adjusted as per response, and finally tapered down slowly.^[12]^ However, cautious usage is advised, as they can inhibit collagen production and epithelial repair, leading to an increased risk of corneal perforation. For example, in PUK associated with RA, topical steroids are not advised as they delay the wound healing process via suppressing fibroblast infiltration. Also, avoiding adverse effects of long-term corticosteroid usage such as glaucoma and cataract is a critical consideration in PUK, especially in cases associated with chronic underlying systemic disease, where a prolonged disease course is expected.^[133,134]^

In comparison with a topical corticosteroid, topical cyclosporine A 2% (CsA) and tacrolimus 0.03% may be a safer and useful option to promote epithelial healing while avoiding nephrotoxicity associated with systemic administration. CsA directly targets T lymphocytes, inhibiting calcineurin, thereby diminishing T cell function and signaling.^[135]^ Although the mechanism of action of CsA makes it a less potent option for inhibiting innate immune effectors in PUK, its safety and efficacy have been reported in severe corneal ulcers and recalcitrant cases of Mooren's ulcer.^[136,137]^

Topical nonsteroidal anti-inflammatory drugs (NSAIDs) (e.g., ketorolac, diclofenac, bromfenac, nepafenac, flurbiprofen) are potential options to alleviate inflammation in PUK; nevertheless, these agents may predispose to silent corneal melting, especially in elderly patients with concurrent ocular surface diseases and RA.^[138]^ Hence, a short course of low potency steroids (e.g., fluorometholone 0.1%, loteprednol etabonate 0.2%) twice or thrice a day may be prescribed over choosing NSAIDs for anti-inflammatory effect in cases of PUK with RA and a dry ocular surface, along with aggressive topical lubrication.^[12]^

Progestins have been cited as possible topical therapeutic agents for PUK given their ability to downregulate ocular surface inflammation. Medroxyprogesterone 1% binds to glucocorticoid receptors, through which it can exert anti-inflammatory features. Additionally, it can promote collagen synthesis in the cornea by inhibiting neutrophil-related collagenases. Based on these characteristics, medroxyprogesterone has been used, in association with other collagenase inhibitors, to decrease the stromal degradation in instances of corneal thinning and keratolysis.^[139,140]^ However, there is no strong data to support its efficacy specifically in PUK.

Furthermore, adjunct therapy with cycloplegics, along with pressure-lowering medications as required, may be of great help in reducing morbidity.^[12]^

### Systemic Treatment

Systemic therapy should be targeting both reducing the sight and life-threatening complications of the underlying systemic disease. The choice of drug(s) depends on the etiology, clinical presentation, severity of disease, and the suitability of the route of drug administration to the patient.

### Systemic therapy to reduce ocular morbidity

Oral doxycycline 100 mg twice daily antagonizes local collagenolysis via irreversible inhibition of MMP through chelation of the catalytically and structurally active metal ions.^[141]^ It may also help regeneration of a stable stratified epithelium by suppressing scar tissue formation through retarding the migratory keratocytes or fibroblasts.^[141]^ In cases of peripheral corneal melting, oral ascorbic acid 1000 mg daily is added. Therapeutic effects of ascorbic acid on promoting corneal epithelial healing and reducing postkeratitic scarring have been reported.^[142,143]^ In cases of associated scleritis, oral NSAIDs may be added to alleviate the pain and inflammation.^[12]^

### Systemic therapy to reduce mortality from underlying systemic disease

The presence of PUK and necrotizing scleritis in autoimmune diseases highlight a fatal form of systemic vasculitis and requires aggressive systemic immunosuppressant therapy.^[144,145]^ Immunosuppressenat therapy has been proved to reduce mortality in patients with PUK and necrotizing scleritis.^[146]^ Foster and colleagues reported the mean time to death in subjects with PUK and scleritis due to various underlying conditions (e.g., RA, GPA, SLE) to be 24.7 years in patients on systemic immunosuppressant therapy versus 10.7 years in those without.^[146,147]^ Since systemic immunosuppressant agents are often indicated for PUK, it can be helpful to coordinate care with a rheumatologist experienced in using these drugs and handling their adverse effects. Detailed knowledge of different systemic immunosuppressive drugs used for autoimmune diseases may be out of the scope of an ophthalmologist's practice. However, a basic understanding of these drugs, their mechanisms, and their adverse effects are helpful for any clinician involved in treating PUK patients. Table 4 summarizes important adverse effects and critical alarming signs of these drugs that an ophthalmologist recognizes during follow-up of PUK [Table 4]. Moreover, in cases of pandemics due to viral agents, e.g., COVID-19, immunosuppression may predispose to lethal opportunistic infections.^[148]^ This risk must be discussed with the patient at the time of starting therapy.

### Nonimmunomodulatory agents 

Traditionally, systemic corticosteroid therapy has been the first-line therapy for acute PUK. Although many other immunosuppressive agents have been proposed for treating immune conditions, the availability of corticosteroids and their rapid therapeutic effect have solidified them as a mainstay in treating severe inflammatory diseases. Corticosteroids, usually intravenous methylprednisolone or oral prednisone, are administered with a starting dosage of 1 mg/kg/day, followed by tapering based on clinical response. Systemic corticosteroid therapy should never be delayed in cases with advanced corneal thinning and impending danger of permanent vision loss, and higher pulse doses (1 g/day IV methylprednisolone) may be used in severe cases.^[1,146,149]^

Long-term adverse effects of systemic steroids, in association with their documented failure of controlling systemic vasculitis in monotherapy regimens, often necessitates the concurrent use of systemic immunomodulatory agents. Additionally, in patients with a worsening clinical course on corticosteroids or those who are unable to be tapered off of high-dose steroid therapy for longer than one month, an immunomodulatory agent should always be considered.^[150]^

### Immunomodulatory Agents

Immunosuppressive agents available for use in ocular inflammatory conditions include antimetabolites, T-cell inhibitors, biologic agents, and alkylating agents.^[151]^ The choice of agent usually depends on the underlying systemic disease [Table 3].

Among antimetabolites, methotrexate, azathioprine, and mycophenolate mofetil have been widely used to treat PUK.^[150]^ In patients with RA-associated PUK unresponsive to systemic corticosteroid therapy, methotrexate and azathioprine were found to be effective.^[152]^ Azathioprine is a purine nucleoside analog, which interferes with DNA replication and RNA transcription, primarily targeting B and T lymphocytes.^[153]^ Systemic azathioprine has been shown to control the ocular immune disease with a mean oral dose of 2 mg/kg/day.^[78,150]^ Methotrexate is a folic acid analog inhibiting the production of thymidylate, an essential molecule necessary for DNA replication. Similar to azathioprine, methotrexate targets rapidly proliferating cells, such as peripheral lymphocytes. An oral regimen of methotrexate with a dosage of 7.5–25 mg/week has been reported to be effective in treating immune corneal ulcers.^[145]^ Mycophenolate mofetil, a selective inhibitor of guanosine nucleotide synthesis, appears to be safer and more effective for treating PUK compared to methotrexate and azathioprine.^[154,155]^ Particularly in cases where adverse effects of other drugs are not tolerable, mycophenolate mofetil is a viable option with an oral dosage of 1 g twice daily.^[152]^

Within the category of T-cell inhibitors, systemic cyclosporin A has been proposed as an effective initial immunosuppressant treatment for ocular immune conditions. However, it is generally not as useful for the treatment of severe systemic inflammatory conditions compared to antimetabolites and alkylating agents, so it is a more appropriate option in PUK without underlying systemic vasculitis. It does carry a risk of nephrotoxicity, and so caution should be taken in patients with underlying renal dysfunction.^[156]^

The alkylating agents, including cyclophosphamide and chlorambucil, are reserved for immune conditions unresponsive to antimetabolites and corticosteroids. These agents cause irreversible DNA crosslinking, leading to apoptosis in rapidly dividing cells such as T lymphocytes. They have been reported to be highly effective in persistent PUK cases.^[144]^ Cyclophosphamide may be administered orally (1–2 m/kg/day) or intravenously (monthly pulses) for destructive ocular immune conditions. In a small case series, it was well-tolerated and effective and controlling both ocular disease and underlying systemic vasculitis.^[157]^

One class of biologic agents that have been used to treat PUK are anti-TNF drugs, including etanercept, infliximab, adalimumab, and golimumab. Etanercept is a decoy receptor for TNF, while the other three agents are monoclonal anti-TNF antibodies. TNF-α is a proinflammatory cytokine released by macrophages and other immune cells that have been targeted by therapy in RA and other autoimmune conditions. Anti-TNF drugs can inhibit both the activity of TNF-α and the production of MMPs, halting the progression of immune keratolysis in PUK.^[158,159]^ These agents have been successful in treating PUK, uveitis, and scleritis associated with immune diseases. ${}^{\left[160--162\right]}$ Infliximab has shown success in halting corneal thinning, but there are reports of resistant disease, as well as severe cardiopulmonary side effects such as myocardial infarcation and pulmonary embolism.^[163]^ Etanercept is less efficacious than infliximab and has been associated with drug-induced scleritis, which has limited its usage in ocular immune conditions.^[160]^ Accordingly, adalimumab has been proposed as an effective anti-TNF agent with acceptable safety and efficacy profile.^[164]^ However, drug-induced corneal infiltrate has been reported in patients undergoing adalimumab therapy, which may limit its usage for ocular conditions in the future.^[165]^

Rituximab, another biologic immunomodulatory agent, is a monoclonal antibody against CD20, which is expressed on the surface of B lymphocytes. Although the role of B cells and humoral immunity in the pathogenesis of PUK is not well understood, anti-B cell therapy with rituximab has been described.^[166]^ It has been used to treat uveitis, scleritis, and refractory PUK associated with GPA, and the results have been promising.^[167,168]^ A recent small case series also reported success in the treatment of RA-associated PUK, with excellent control of ocular inflammation, and also improvement in the articular and vasculitic disease.^[169]^ In addition to rituximab, tocilizumab, and belimumab are non-anti TNF antibodies to IL-6R and BAFF, respectively, that have been used sparingly but successfully in the treatment of PUK. In one case series of patients treated with rituximab, tocilizumab, or belimumab, these agents were found to be more effective than anti-TNF agents for treatment-resistant PUK.^[170]^

Combination therapy with immunosuppressive agents is a common approach in treating systemic and ocular immune conditions. The main limitation in combination therapy is the cumulative risk of adverse effects. The most common combination regimens include an immunomodulatory agent with a systemic corticosteroid, to enhance therapeutic effect and facilitate steroid tapering.^[150]^ The synergistic effect of combined therapy with antimetabolites and corticosteroids has been reported in treating ocular immune conditions.^[171]^ The combination of antimetabolites and cyclosporine has been used widely as an anti-transplant rejection regimen and has been proposed for use in ocular conditions with an acceptable safety profile. Although cyclophosphamide is frequently combined with corticosteroids for treating systemic vasculitis, the increased risk of opportunistic infections and malignancies has limited its use in conjunction with biologic or other immunomodulatory therapies.^[150]^ Recently, Dominguez-Casas and colleagues reported that 48% of patients who were initially started on an anti-TNF alpha agent had to be switched to another biological agent because of treatment failure or adverse effects.^[170]^ Biotherapy in most of their cohort achieved a rapid and maintained improvement in the efficacy outcome parameters after three months of starting therapy.^[170]^

Over the last 10 years, drugs known as Janus kinase (JAK) inhibitors have been reported in various autoimmune diseases, such as RA, psoriatic arthritis, ulcerative colitis, and others. In addition, JAK inhibitors seem to be effective regimens for intractable noninfectious inflammatory eye disease, including uveitis and scleritis.^[172]^ Furthermore, novel therapeutic agents, e.g., CacicolⓇ (a matrix regenerating agent) are being investigated.^[173]^ Sevik and colleagues reported complete epithelial healing in 86.9% of their case series with persistent epithelial defect refractory to conventional therapy after 2 to 20 days of treatment.^[173]^

### Surgical Management

Along with systemic immunosuppression, surgical interventions are often required in PUK with severe corneal thinning or corneal perforations for tectonic reconstruction.^[151]^ Systemic immunosuppression is mandatory to reduce graft melts, rejection rates, and PUK recurrences.^[12]^ Early interventions with adequate immunosuppression have been reported to restore the integrity of the globe in 92% of cases.^[144]^ In addition, the six-month graft survival rates after PK ranged from 20 to 40%.^[144,174]^ Available options include tissue adhesives, bandage contact lens, penetrating or lamellar graft, and amniotic membrane transplantation (AMT).^[12]^ The choice of the intervention depends on the extent of the disease and the size of perforation.

The use of tissue adhesives, followed by the application of a contact lens, is a straightforward and widely available option for the management of descemetocele or perforations smaller than 2–3 mm.^[175,176]^ Cyanoacrylate tissue adhesives are tolerable and effective in managing immune corneal ulcers, such as PUK associated with systemic diseases. The possibility of treatment failure, potential infectious complications, and patient discomfort following the application of tissue adhesives necessitate close follow-up.^[177]^ Reinforcement of corneal tissue with these adhesives often serves as a bridge to further surgical intervention. For perforations 3–5 mm, a tuck-in Tenon patch graft may be a useful option.^[178]^

The conjunctiva adjacent to the peripheral ulcer serves as a reservoir of immune cells, inflammatory cytokines, and proteolytic enzymes. Accordingly, resection of the perilimbal conjunctiva has been proposed as a therapeutic intervention to promote the resolution of inflammation. However, this treatment is limited by a recurrence of the immune response as the conjunctiva grows back to the adjacent corneal ulcer. Evidence on the efficacy of conjunctival resection is limited due to a small number of cases and short follow-up periods. Therefore, the treatment is controversial. A recent report of three patients indicated that conjunctival resection could promote PUK resolution and decrease the need for systemic immunosuppressive therapy, however, larger studies are needed to confirm the results of these clinical observations.^[179]^

In conjunction with appropriate systemic treatment, corneal grafts can preserve the integrity of eyes with impending corneal perforation.^[180]^ PK with large-diameter grafts (9–9.5 mm) may be needed to remove the inflamed peripheral cornea, but it is associated with a higher risk of graft rejection due to the presence of the adjacent limbal vasculature. Large-diameter grafts are associated with a two-times higher risk of rejection compared to average-sized PK.^[181,182]^ This risk can even be higher in PUK cases with active underlying inflammation, as Maneo et al reported that keratoplasty performed for PUK had the highest probability of rejection requiring regrafting compared to PK for other indications.^[183]^ Graft survival rates of < 40% at six months have been reported in PK for PUK.^[184]^ To reduce the risk of graft rejection, small-diameter tectonic grafts ranging from 3 to 5.5 mm can be used, but they are associated with poorer visual outcomes.^[182]^

As an alternative to PK, crescent-shaped, “match and patch” lamellar keratoplasty has become a commonly used technique in PUK.^[182]^ Lamellar techniques provide some advantages over a penetrating graft in inflammatory corneal ulcers. First, the risk of graft rejection is lower for a lamellar graft. Second, it avoids an intraocular procedure (if perforation has not yet occurred), reducing the risk of endophthalmitis, glaucoma, or cataract. Finally, by adding to the thickness of the cornea, the risk of recurrent corneal perforation decreases in lamellar grafts, which can be a great advantage in cases of ongoing inflammation, such as in PUK or Mooren's ulcers.^[185]^

The crescentic lamellar keratoplasty technique consists of placing a ring-shaped lamellar graft on the peripheral cornea and suturing it to the host bed. The extent of the ulcer determines the size and shape of the graft.^[182]^ As a modification to this technique in cases of perforation, double-layer peripheral keratoplasty has been proposed for immune-mediated corneal thinning in Mooren's ulcers. A posterior corneal patch graft is placed under the crescent-shaped lamellar graft, reducing the rate of ulcer recurrence from 26% to 10% with successful visual outcomes.^[186]^ In addition to crescentic lamellar grafts, a decentered large-diameter superficial anterior lamellar keratoplasty (SALK) has also been used in PUK with a successful outcome.^[187]^

AMT can also be used in PUK cases, having the dual effect of reducing inflammation of the underlying cornea and providing mechanical support. Expression of Fas ligand and activation of suppressor T cells in AMT was reported to be associated with anti-inflammatory properties.^[188]^ AMT has been also been shown to decrease the corneal inflammatory response via apoptosis of innate immune cells.^[189]^ Beyond its anti-inflammatory effects, AMT has been useful as a support for the physical corneal structure to reduce the risk of perforation in peripheral inflammatory and noninflammatory ulcers.^[190,191]^ Although few studies have evaluated the role of AMT in treating PUK, this technique has been widely used for Mooren's ulcers. In two case series, AMT reduced pain symptoms and stabilized vision in up to 50% of patients, although it occasionally required multiple applications.^[192,193]^ Additionally, lamellar keratoplasty combined with AMT is becoming a popular surgical option for perforations < 4 mm.^[194]^ Lastly, the option of keratoprosthesis is also being explored for severe cases.^[195]^

In conclusion, PUK is a rare but serious ocular disease with important systemic implications. The underlying autoimmune pathogenesis is not fully understood but appears to involve both cell-mediated and auto-antibody-mediated components, resulting in the breakdown of peripheral corneal tissue. Reducing ocular morbidity through topical and surgical methods, in addition to controlling systemic inflammation with immunomodulatory therapy, is paramount to successful treatment; emerging biologic therapies and surgical techniques have shown promise.

##  Financial Support and Sponsorship 

None.

##  Conflicts of Interest 

None declared.

## References

[B1] Tauber J, Sainz de la Maza M, Hoang-Xuan T, Foster CS (1990). An analysis of therapeutic decision making regarding immunosuppressive chemotherapy for peripheral ulcerative keratitis. Cornea.

[B2] McKibbin M, Isaacs JD, Morrell AJ (1999). Incidence of corneal melting in association with systemic disease in the Yorkshire Region, 1995-7. Br J Ophthalmol.

[B3] Robin JB, Schanzlin DJ, Verity SM, Barron BA, Arffa RC, Suarez E, et al (1986). Peripheral corneal disorders. Surv Ophthalmol.

[B4] Müller LJ, Pels E, Schurmans LR, Vrensen GF (2004). A new three-dimensional model of the organization of proteoglycans and collagen fibrils in the human corneal stroma. Exp Eye Res.

[B5] Messmer EM, Foster CS (1995). Destructive corneal and scleral disease associated with rheumatoid arthritis. Medical and surgical management Cornea.

[B6] Marsovszky L, Resch MD, Németh J, Toldi G, Medgyesi E, Kovács L, et al (2013). In vivo confocal microscopic evaluation of corneal Langerhans cell density, and distribution and evaluation of dry eye in rheumatoid arthritis. Innate Immun.

[B7] Gipson IK, Hori Y, Argüeso P (2004). Character of ocular surface mucins and their alteration in dry eye disease. Ocul Surf.

[B8] Müller L, Vrensen G, Pels L, Cardozo BN, Willekens B (1997). Architecture of human corneal nerves. Invest Ophthalmol Vis Sci.

[B9] Gomes BF, Santhiago MR (2021). Biology of peripheral ulcerative keratitis. Exp Eye Res.

[B10] Timlin HM, Hall HN, Foot B, Koay P (2018). Corneal perforation from peripheral ulcerative keratopathy in patients with rheumatoid arthritis: epidemiological findings of the British Ophthalmological Surveillance Unit. Br J Ophthalmol.

[B11] John SL, Morgan K, Tullo AB, Holt PJ (1992). Corneal autoimmunity in patients with peripheral ulcerative keratitis (PUK) in association with rheumatoid arthritis and Wegener's granulomatosis. Eye.

[B12] Gupta Y, Kishore A, Kumari P, Balakrishnan N, Lomi N, Gupta N, et al (2021). Peripheral ulcerative keratitis. Surv Ophthalmol.

[B13] Tavassoli S, Wong N, Chan E (2021). Ocular manifestations of rosacea: a clinical review. Clin Exp Ophthalmol.

[B14] Sharma N, Sinha G, Shekhar H, Titiyal JS, Agarwal T, Chawla B, et al (2015). Demographic profile, clinical features and outcome of peripheral ulcerative keratitis: a prospective study. Br J Ophthalmol.

[B15] Sainz de la Maza M, Foster CS, Jabbur NS, Baltatzis S (2002). Ocular characteristics and disease associations in scleritis-associated peripheral keratopathy. Arch Ophthalmol.

[B16] Watson PG, Bovey E (1985). Anterior segment fluorescein angiography in the diagnosis of scleral inflammation. Ophthalmology.

[B17] Watson PG (1990). Vascular changes in peripheral corneal destructive disease. Eye.

[B18] Gomes BAF, Santhiago MR, Jorge PA, et al (2015). Corneal involvement in systemic inflammatory diseases. Eye Contact Lens.

[B19] Jones RR, Maguire LJ (1992). Corneal complications after cataract surgery in patients with rheumatoid arthritis. Cornea.

[B20] Chanbour W, Mokdad I, Mouhajer A, Jarade E (2019). Late-onset sterile peripheral ulcerative keratitis post-corneal collagen crosslinking. Cornea.

[B21] Messmer EM, Foster CS (1999). Vasculitic peripheral ulcerative keratitis. Surv Ophthalmol.

[B22] Allansmith MR, McClellan BH (1975). Immunoglobulins in the human cornea. Am J Ophthalmol.

[B23] Mondino BJ, Brady KJ (1981). Distribution of hemolytic complement in the normal cornea. Arch Ophthalmol.

[B24] Mondino BJ (1988). Inflammatory diseases of the peripheral cornea. Ophthalmology.

[B25] Dana MR, Qian Y, Hamrah P (2000). Twenty-five-year panorama of corneal immunology: emerging concepts in the immunopathogenesis of microbial keratitis, peripheral ulcerative keratitis, and corneal transplant rejection. Cornea.

[B26] Romagnani S, Parronchi P, D'Elios MM, Romagnani P, Annunziato F, Piccinni MP, et al (1997). An update on human Th1 and Th2 cells. Int Arch Allergy Immunol.

[B27] Rao DA, Gurish MF, Marshall JL, Slowikowski K, Fonseka CY, Liu Y, et al (2017). Pathologically expanded peripheral T helper cell subset drives B cells in rheumatoid arthritis. Nature.

[B28] Martinez Valenzuela L, Bordignon Draibe J, Fulladosa Oliveras X, Matamoros OB, Garrit JMC, Ambrós JT (2019). T-lymphocyte in ANCA-associated vasculitis: what do we know? A pathophysiological and therapeutic approach. Clin Kidney J.

[B29] Giscombe R, Nityanand S, Lewin N, Grunewald J, Lefvert AK (1998). Expanded T cell populations in patients with Wegener's granulomatosis: characteristics and correlates with disease activity. J Clin Immunol.

[B30] Mondino BJ, Brown SI, Rabin BS (1978). Cellular immunity in Mooren's ulcer. Am J Ophthalmol.

[B31] Foster CS, Kenyon KR, Greiner J, Greineder DK, Friedland B, Allansmith MR (1979). The immunopathology of Mooren's ulcer. Am J Ophthalmol.

[B32] Zhao JC, Jin XY (1993). Immunological analysis and treatment of Mooren's ulcer with cyclosporin A applied topically. Cornea.

[B33] Nogueira E, Hamour S, Sawant D, Henderson S, Mansfield N, Chavele K-M, et al (2010). Serum IL-17 and IL-23 levels and autoantigen-specific Th17 cells are elevated in patients with ANCA-associated vasculitis. Nephrol Dial Transplant.

[B34] Tarzi RM, Pusey CD (2014). Current and future prospects in the management of granulomatosis with polyangiitis (Wegener's granulomatosis). Ther Clin Risk Manag.

[B35] Shiuey Y, Foster CS (1998). Peripheral ulcerative keratitis and collagen vascular disease. Int Ophthalmol Clin.

[B36] Nagata S, Glovsky MM, Kunkel SL (1987). Anaphylatoxin-induced neutrophil chemotaxis and aggregation. Limited aggregation and specific desensitization induced by human C3a and synthetic C3a octapeptides Int Arch Allergy Appl Immunol.

[B37] Metzemaekers M, Gouwy M, Proost P (2020). Neutrophil chemoattractant receptors in health and disease: double-edged swords. Cell Mol Immunol.

[B38] Quintana LF, Kronbichler A, Blasco M, Zhao M-H, Jayne D (2019). ANCA associated vasculitis: The journey to complement-targeted therapies. Mol Immunol.

[B39] Albert DM, Jakobiec FA (2000). Principles and practice of ophthalmology.

[B40] Morgan-Warren PJ, Dulku S, Ravindran J, Smith G (2014). Peripheral ulcerative keratitis as the presenting feature of systemic rheumatoid vasculitis without joint involvement. Int Ophthalmol.

[B41] Squatrito D, Emmi G, Silvestri E, Ciucciarelli L, D'Elios MM, Prisco D, et al (2014). Pathogenesis and potential therapeutic targets in systemic lupus erythematosus: from bench to bedside. Auto Immun Highlights.

[B42] Reynolds I, John SL, Tullo AB, Ayad S, Morgan K, Ballardie FW, et al (1998). Characterization of two corneal epithelium-derived antigens associated with vasculitis. Invest Ophthalmol Vis Sci.

[B43] Albers FW, Majoor MH, Van der Gaag R (1992). Corneal autoimmunity in a patient with relapsing polychondritis. Eur Arch Otorhinolaryngol.

[B44] Mondino BJ, Brown SI, Rabin BS (1978). Autoimmune phenomena of the external eye. Ophthalmology.

[B45] Gottsch JD, Liu SH, Minkovitz JB, Goodman DF, Srinivasan M, Stark WJ (1995). Autoimmunity to a cornea-associated stromal antigen in patients with Mooren's ulcer. Invest Ophthalmol Vis Sci.

[B46] Rodeghero R, Cao Y, Olalekan SA, Iwakua Y, Glant TT, Finnegan A (2013). Location of CD4+ T cell priming regulates the differentiation of Th1 and Th17 cells and their contribution to arthritis. J Immunol.

[B47] Bugatti S, Vitolo B, Caporali R, Montecucco C, Manzo A (2014). B cells in rheumatoid arthritis: from pathogenic players to disease biomarkers. Biomed Res Int.

[B48] Eghrari AO, Riazuddin SA, Gottsch JD (2015). Overview of the cornea: structure, function, and development. Prog Mol Biol Transl Sci.

[B49] Jamerson EC, Elhusseiny AM, ElSheikh RH, Eleiwa TK, El Sayed YM

[B50] Watanabe R, Ishii T, Yoshida M, Takada N, Yokokura S, Shirota Y, et al (2017). Ulcerative keratitis in patients with rheumatoid arthritis in the modern biologic era: a series of eight cases and literature review. Int J Rheum Dis.

[B51] Artifoni M, Rothschild PR, Brézin A, Guillevin L, Puéchal X (2014). Ocular inflammatory diseases associated with rheumatoid arthritis. Nat Rev Rheumatol.

[B52] Visse R, Nagase H (2003). Matrix metalloproteinases and tissue inhibitors of metalloproteinases: structure, function, and biochemistry. Circ Res.

[B53] Brejchova K, Liskova P, Cejkova J, Jirsova K (2010). Role of matrix metalloproteinases in recurrent corneal melting. Exp Eye Res.

[B54] Smith VA, Rishmawi H, Hussein H, Easty DL (2001). Tear film MMP accumulation and corneal disease. Br J Ophthalmol.

[B55] Smith VA, Hoh HB, Easty DL (1999). Role of ocular matrix metalloproteinases in peripheral ulcerative keratitis. Br J Ophthalmol.

[B56] Yagci A (2012). Update on peripheral ulcerative keratitis. Clin Ophthalmol.

[B57] Gregory JK, Foster CS (1996). Peripheral ulcerative keratitis in the collagen vascular diseases. Int Ophthalmol Clin.

[B58] Bouchard CS, Meyer MA, McDonnell JF (1997). Bilateral peripheral ulcerative keratitis associated with pyoderma gangrenosum. Cornea.

[B59] García M, Garrido G, Ortiz A, et al (2015). AB0370 Peripheral ulcerative keratitis associated with autoimmune diseases: a typical ocurrence in the biologic era. Ann Rheum Dis.

[B60] Pelegrín L, Hernández-Rodríguez J, Torras J, et al (2015). AB0678 Peripheral ulcerative keratitis associated to autoimmune systemic diseases: visual prognosis and occurrence while systemic disease in remission. Ann Rheum Dis.

[B61] Levitt AE, McManus KT, McClellan AL, Davis JL, Goldhardt R, Galor A (2015). Ocular inflammation in the setting of concomitant systemic autoimmune conditions in an older male population. Cornea.

[B62] Cao Y, Zhang W, Wu J, Zhang H, Zhou H (2017). Peripheral ulcerative keratitis associated with autoimmune disease: pathogenesis and treatment. J Ophthalmol.

[B63] Galor A, Thorne JE

[B64] Fernandes SR, Singsen BH, Hoffman GS (2000). Sarcoidosis and systemic vasculitis. Semin Arthritis Rheum.

[B65] Siracuse-Lee D, Saffra N (2006). Peripheral ulcerative keratitis in sarcoidosis: a case report. Cornea.

[B66] Harthan JS, Reeder RE (2013). Peripheral ulcerative keratitis in association with sarcoidosis. Cont Lens Anterior Eye.

[B67] Ji YS, Yoon KC (2014). A rare case of peripheral ulcerative keratitis associated with Behçet's disease. Int Ophthalmol.

[B68] Mady R, Grover W, Butrus S

[B69] Tan MH, Chen SD, Rubinstein A, Bron AJ (2006). Corneal perforation due to severe peripheral ulcerative keratitis in Crohn disease. Cornea.

[B70] Herbert VG, Lögering B, von Gruben V, Filev F, Klemm M, Reich K (2014). Ulcerative keratitis in psoriasis: a rare variant of psoriatic ocular inflammatory disease. Br J Dermatol.

[B71] Mahmood MA, Pillai S, Limaye SR (1991). Peripheral ulcerative keratitis associated with hideradenitis suppurativa. Cornea.

[B72] Meskin SW, Carlson EM (2011). Mooren's-type ulceration associated with severe hidradenitis suppurativa: a case report and literature review. Ocul Immunol Inflamm.

[B73] Sainz de la Maza M, Foster CS (1994). Peripheral ulcerative keratitis and malignancies. Cornea.

[B74] Malecha MA, Holland EJ (2002). Peripheral keratitis associated with chronic myelomonocytic leukemia. Cornea.

[B75] Morjaria R, Barge T, Mordant D, Elston J (2014). Peripheral ulcerative keratitis as a complication of acute myeloid leukaemia. BMJ Case Rep.

[B76] Fournié P, Malecaze F, Coullet J, Arné JL (2007). Pyoderma gangrenosum with necrotizing sclerokeratitis after cataract surgery. J Cataract Refract Surg.

[B77] Ayyala RS, Armstrong S (1998). Corneal melting and scleromalacia perforans in a patient with pyoderma gangrenosum and acute myeloid leukemia. Ophthalmic Surg Lasers.

[B78] Brown BA, Parker CT, Bower KS (2001). Effective steroid-sparing treatment for peripheral ulcerative keratitis and pyoderma gangrenosum. Cornea.

[B79] Miserocchi E, Modorati G, Foster CS, Brancato R (2002). Ocular and extracutaneous involvement in pyoderma gangrenosum. Ophthalmology.

[B80] Arora T, Sharma N, Shashni A, Titiyal JS (2015). Peripheral ulcerative keratitis associated with chronic malabsorption syndrome and miliary tuberculosis in a child. Oman J Ophthalmol.

[B81] Rafiezadeh P, Schmack I, Shajari M, Kohnen T (2018). Autoimmune keratitis in mycobacterium tuberculosis. J Curr Ophthalmol.

[B82] Kamal S, Kumar R, Kumar S, Goel R

[B83] Ploysangam P, Mattern RM (2019). Perforating peripheral ulcerative keratitis in syphilis. Case Rep Ophthalmol.

[B84] Vignesh AP, Srinivasan R, Vijitha S (2016). Ocular syphilis masquerading as bilateral peripheral ulcerative keratitis. Taiwan J Ophthalmol.

[B85] Prasher P, Di Pascuale M, Cavanagh HD (2008). Bilateral chronic peripheral ulcerative keratitis secondary to cat-scratch disease. Eye Contact Lens.

[B86] Tavassoli S, Gunn D, Tole D, Darcy K (2019). Peripheral ulcerative keratitis with corneal melt as the primary presentation in a case of human immunodeficiency virus. BMJ Case Rep.

[B87] Soni ND, Ingole AB, Murade SM (2013). An unusual case of peripheral ulcerative keratitis as a presenting feature in an otherwise healthy patient with undiagnosed human immunodeficiency virus infection and low CD4 counts. Indian J Ophthalmol.

[B88] Gharai S, Venkatesh P, Tandon R, Garg S (2007). Peripheral ulcerative keratitis and central retinal vein occlusion as the initial manifestation of HIV infection. Ocul Immunol Inflamm.

[B89] Chetty R (2001). Vasculitides associated with HIV infection. J Clin Pathol.

[B90] Du Toit SH, Smit DP (2014). Mooren's ulcer of the cornea after immune reconstitution. Aids.

[B91] Wei DW, Pagnoux C, Chan CC (2017). Peripheral ulcerative keratitis secondary to chronic hepatitis B infection. Cornea.

[B92] Wilson SE, Lee WM, Murakami C, Weng J, Moninger GA (1994). Mooren-type hepatitis C virus-associated corneal ulceration. Ophthalmology.

[B93] Kedhar SR, Belair ML, Jun AS, Sulkowski M, Thorne JE (2007). Scleritis and peripheral ulcerative keratitis with hepatitis C virus-related cryoglobulinemia. Arch Ophthalmol.

[B94] Enomoto M, Nakanishi T, Ishii M, Tamori A, Kawada N (2008). Entecavir to treat hepatitis B-associated cryoglobulinemic vasculitis. Ann Intern Med.

[B95] Guillevin L, Mahr A, Callard P, Godmer P, Pagnoux C, Leray E, et al (2005). Hepatitis B virus-associated polyarteritis nodosa: clinical characteristics, outcome, and impact of treatment in 115 patients. Medicine.

[B96] Mondino BJ, Hofbauer JD, Foos RY (1987). Mooren's ulcer after penetrating keratoplasty. Am J Ophthalmol.

[B97] Kiire CA, Srinivasan S, Inglis A (2011). Peripheral ulcerative keratitis after cataract surgery in a patient with ocular cicatricial pemphigoid. Cornea.

[B98] Burkholder BM, Kuo IC (2016). Peripheral ulcerative keratitis following laser in situ keratomileusis. Case Rep Ophthalmol.

[B99] Díaz-Valle D, Benítez del Castillo JM, Castillo A, Sayagués O, Bañares A, García-Sánchez J (1998). Immunologic and clinical evaluation of postsurgical necrotizing sclerocorneal ulceration. Cornea.

[B100] Akpek EK, Demetriades AM, Gottsch JD (2000). Peripheral ulcerative keratitis after clear corneal cataract extraction(1). J Cataract Refract Surg.

[B101] Salamon SM, Mondino BJ, Zaidman GW (1982). Peripheral corneal ulcers, conjunctival ulcers, and scleritis after cataract surgery. Am J Ophthalmol.

[B102] Ladas JG, Mondino BJ (2000). Systemic disorders associated with peripheral corneal ulceration. Curr Opin Ophthalmol.

[B103] Keenan JD, Mandel MR, Margolis TP (2011). Peripheral ulcerative keratitis associated with vasculitis manifesting asymmetrically as fuchs superficial marginal keratitis and terrien marginal degeneration. Cornea.

[B104] Odorcic S, Keystone EC, Ma JJ (2009). Infliximab for the treatment of refractory progressive sterile peripheral ulcerative keratitis associated with late corneal perforation: 3-year follow-up. Cornea.

[B105] Riley GP, Harrall RL, Watson PG, Cawston TE, Hazleman BL (1995). Collagenase (MMP-1) and TIMP-1 in destructive corneal disease associated with rheumatoid arthritis. Eye.

[B106] Mannis  M, Holland E

[B107] Cohn H, Mondino BJ, Brown SI, Hall GD (1979). Marginal corneal ulcers with acute beta streptococcal conjunctivitis and chronic dacryocystitis. Am J Ophthalmol.

[B108] Taylor PB, Tabbara KF (1986). Peripheral corneal infections. Int Ophthalmol Clin.

[B109] Awais M, Anwar MI, Iftikhar R, Iqbal Z, Shehzad N, Akbar B (2015). Rosacea – the ophthalmic perspective. Cutan Ocul Toxicol.

[B110] Suzuki T, Kinoshita S

[B111] Suzuki T

[B112] Suzuki T

[B113] Mondino BJ, Brown SI, Mondzelewski JP (1978). Peripheral corneal ulcers with herpes zoster ophthalmicus. Am J Ophthalmol.

[B114] Kim HB, Ostler HB (1977). Marginal corneal ulcer due to beta-streptococcus. Arch Ophthalmol.

[B115] Baum J, Fedukowicz HB, Jordan A (1980). A survey of Moraxella corneal ulcers in a derelict population. Am J Ophthalmol.

[B116] Hutton WL, Sexton RR (1972). Atypical Pseudomonas corneal ulcers in semicomatose patients. Am J Ophthalmol.

[B117] Baijal V, Palit MG, Choudhary T, Gurunadh VS, Shanker S (1999). Terrien's marginal degeneration: case report. Med J Armed Forces India.

[B118] Wilson SE, Lin DT, Klyce SD, Insler MS (1990). Terrien's marginal degeneration: corneal topography. Refract Corneal Surg.

[B119] Ding Y, Murri MS, Birdsong OC, Ronquillo Y, Moshirfar M (2019). Terrien marginal degeneration. Surv Ophthalmol.

[B120] Rishi P, Shields CL, Eagle RC (2014). Conjunctival intraepithelial neoplasia with corneal furrow degeneration. Indian J Ophthalmol.

[B121] Rumelt S, Rehany U (1997). Computerized corneal topography of furrow corneal degeneration. J Cataract Refract Surg.

[B122] Accorinti M, Gilardi M, Giubilei M, De Geronimo D, Iannetti L (2014). Corneal and scleral dellen after an uneventful pterygium surgery and a febrile episode. Case Rep Ophthalmol.

[B123] Fresina M, Campos EC (2009). Corneal 'dellen' as a complication of strabismus surgery. Eye.

[B124] Sharma B, Bajoria SK, Patnaik A, Barbhaya R

[B125] Kafkala C, Choi J, Zafirakis P, Baltatzis S, Livir-Rallatos C, Rojas B (2006). Mooren ulcer: an immunopathologic study. Cornea.

[B126] Zelefsky JR, Srinivasan M, Kundu A, Lietman T, Whitcher JP, Wang K, et al (2007). Hookworm infestation as a risk factor for Mooren's ulcer in South India. Ophthalmology.

[B127] Zegans ME, Srinivasan M (1998). Mooren's Ulcer. Int Ophthalmol Clin.

[B128] Srinivasan M, Zegans ME, Zelefsky JR, Kundu A, Lietman T, Whitcher JP, et al (2007). Clinical characteristics of Mooren's ulcer in South India. Br J Ophthalmol.

[B129] Watson PG (1997). Management of Mooren's ulceration. Eye.

[B130] Alhassan MB, Rabiu M, Agbabiaka IO (2014). Interventions for Mooren's ulcer. Cochrane Database Syst Rev.

[B131] Thring TSA, Hili P, Naughton DP (2009). Anti-collagenase, anti-elastase and anti-oxidant activities of extracts from 21 plants. BMC Complement Altern Med.

[B132] Ramaesh T, Ramaesh K, Riley SC, West JD, Dhillon B (2012). Effects of N-acetylcysteine on matrix metalloproteinase-9 secretion and cell migration of human corneal epithelial cells. Eye.

[B133] Perry HD, Golub LM (1985). Systemic tetracyclines in the treatment of noninfected corneal ulcers: a case report and proposed new mechanism of action. Ann Ophthalmol.

[B134] Ralph RA (2000). Tetracyclines and the treatment of corneal stromal ulceration: a review. Cornea.

[B135] Kaçmaz RO, Kempen JH, Newcomb C, Daniel E, Gangaputra S, Nussenblatt RB, et al (2010). Cyclosporine for ocular inflammatory diseases. Ophthalmology.

[B136] Tandon R, Chawla B, Verma K, Sharma N, Titiyal JS (2008). Outcome of treatment of Mooren ulcer with topical cyclosporine A 2%. Cornea.

[B137] Zierhut M, Thiel HJ, Weidle EG, Waetjen R, Pleyer U (1989). Topical treatment of severe corneal ulcers with cyclosporin A. Graefes Arch Clin Exp Ophthalmol.

[B138] Goodman LS (1996). Goodman and Gilman's the pharmacological basis of therapeutics.

[B139] Khan L, Batavia E (2019). Medroxyprogesterone to treat corneal thinning postcurvularia keratitis. Oman J Ophthalmol.

[B140] Hicks CR, Crawford GJ (2003). Melting after keratoprosthesis implantation: the effects of medroxyprogesterone. Cornea.

[B141] Smith V, Cook S (2004). Doxycycline—a role in ocular surface repair. Br J Ophthalmol.

[B142] Chen J, Lan J, Liu D, Backman LJ, Zhang W, Zhou Q, et al (2017). Ascorbic acid promotes the stemness of corneal epithelial stem/progenitor cells and accelerates epithelial wound healing in the cornea. Stem Cells Transl Med.

[B143] Cho Y-W, Yoo W-S, Kim S-J, Chung I-Y, Seo S-W, Yoo J-M (2014). Efficacy of systemic vitamin C supplementation in reducing corneal opacity resulting from infectious keratitis. Medicine.

[B144] Messmer EM, Foster CS (1995). Destructive corneal and scleral disease associated with rheumatoid arthritis. Medical and surgical management Cornea.

[B145] Squirrell DM, Winfield J, Amos RS (1999). Peripheral ulcerative keratitis 'corneal melt' and rheumatoid arthritis: a case series. Rheumatology.

[B146] Foster CS, Forstot SL, Wilson LA (1984). Mortality rate in rheumatoid arthritis patients developing necrotizing scleritis or peripheral ulcerative keratitis. Effects of systemic immunosuppression Ophthalmology.

[B147] Ogra S, Sims JL, McGhee CNJ, Niederer RL (2020). Ocular complications and mortality in peripheral ulcerative keratitis and necrotising scleritis: the role of systemic immunosuppression. Clin Exp Ophthalmol.

[B148] Monti S, Balduzzi S, Delvino P, Bellis E, Quadrelli VS, Montecucco C (2020). Clinical course of COVID-19 in a series of patients with chronic arthritis treated with immunosuppressive targeted therapies. Ann Rheum Dis.

[B149] Araki-Sasaki K, Katsuta O, Mano H, Nagano T, Nakamura M (2016). The effects of oral and topical corticosteroid in rabbit corneas. BMC Ophthalmol.

[B150] Jabs DA, Rosenbaum JT, Foster CS, Holland GN, Jaffe GJ, Louie JS, et al (2000). Guidelines for the use of immunosuppressive drugs in patients with ocular inflammatory disorders: recommendations of an expert panel. Am J Ophthalmol.

[B151] Tandon R, Galor A, Sangwan V, Manotosh R

[B152] Burska AN, Hunt L, Boissinot M, Strollo R, Ryan BJ, Vital E, et al (2014). Autoantibodies to posttranslational modifications in rheumatoid arthritis. Mediators Inflamm.

[B153] el-Yazigi A, Wahab FA (1993). Pharmacokinetics of azathioprine after repeated oral and single intravenous administration. J Clin Pharmacol.

[B154] Galor A, Jabs DA, Leder HA, Kedhar SR, Dunn JP, Peters 3rd GB, et al (2008). Comparison of antimetabolite drugs as corticosteroid-sparing therapy for noninfectious ocular inflammation. Ophthalmology.

[B155] Thorne JE, Jabs DA, Qazi FA, Nguyen QD, Kempen JH, Dunn JP (2005). Mycophenolate mofetil therapy for inflammatory eye disease. Ophthalmology.

[B156] McCarthy JM, Dubord PJ, Chalmers A, Kassen BO, Rangno KK (1992). Cyclosporine A for the treatment of necrotizing scleritis and corneal melting in patients with rheumatoid arthritis. J Rheumatol.

[B157] Clewes AR, Dawson JK, Kaye S, Bucknall RC (2005). Peripheral ulcerative keratitis in rheumatoid arthritis: successful use of intravenous cyclophosphamide and comparison of clinical and serological characteristics. Ann Rheum Dis.

[B158] Hata M, Nakamura T, Sotozono C, Kumagai K, Kinoshita S, Kurimoto Y (2012). Atypical continuous keratitis in a case of rheumatoid arthritis accompanying severe scleritis. Cornea.

[B159] Iliou C, Anthis N, Tsifetaki N, Kitsos G, Voulgari PV (2012). Clinical images: corneal melt in a woman with longstanding rheumatoid arthritis. Arthritis Rheum.

[B160] Gaujoux-Viala C, Giampietro C, Gaujoux T, Ea H-K, Prati C, Orcel P, et al (2012). Scleritis: a paradoxical effect of etanercept? Etanercept-associated inflammatory eye disease. J Rheumatol.

[B161] Oh JY, Kim MK, Wee WR (2011). Infliximab for progressive peripheral ulcerative keratitis in a patient with juvenile rheumatoid arthritis. Jpn J Ophthalmol.

[B162] Thomas JW, Pflugfelder SC (2005). Therapy of progressive rheumatoid arthritis-associated corneal ulceration with infliximab. Cornea.

[B163] Huerva V, Ascaso FJ, Grzybowski A (2014). Infliximab for peripheral ulcerative keratitis treatment. Medicine.

[B164] Restrepo JP, Medina LF, Molina Mdel P (2015). [Peripheral corneal melting syndrome in psoriatic arthritis treated with adalimumab]. Rev Bras Reumatol.

[B165] Matet A, Daruich A, Beydoun T, Cosnes J, Bourges J-L (2015). Systemic adalimumab induces peripheral corneal infiltrates: a case report. BMC Ophthalmol.

[B166] Tong L, Thumboo J, Tan YK, Wong T-Y, Albani S

[B167] Freidlin J, Wong IG, Acharya N (2007). Rituximab treatment for peripheral ulcerative keratitis associated with Wegener's granulomatosis. Br J Ophthalmol.

[B168] Huerva V, Sanchez MC, Traveset A, Jurjo C, Ruiz A (2010). Rituximab for peripheral ulcerative keratitis with wegener granulomatosis. Cornea.

[B169] Bonnet I, Rousseau A, Duraffour P, Pouchot J, Nguyen CD, Gabison E, et al (2021). Efficacy and safety of rituximab in peripheral ulcerative keratitis associated with rheumatoid arthritis. RMD Open.

[B170] Dominguez-Casas LC, Sánchez-Bilbao L, Calvo-Río V, Maíz O, Blanco A, Beltrán E, et al (2020). Biologic therapy in severe and refractory peripheral ulcerative keratitis (PUK). Multicenter study of 34 patients Semin Arthritis Rheum.

[B171] Pascalis L, Pia G, Aresu G, Frongia T, Barca L (1993). Combined cyclosporin A-steroid-MTX treatment in endogenous non-infectious uveitis. J Autoimmun.

[B172] Paley MA, Karacal H, Rao PK, Margolis TP, Miner JJ (2019). Tofacitinib for refractory uveitis and scleritis. Am J Ophthalmol Case Rep.

[B173] Sevik MO, Turhan SA, Toker E (2018). Topical treatment of persistent epithelial defects with a matrix regenerating agent. J Ocul Pharmacol Ther.

[B174] Bernauer W, Ficker LA, Watson PG, Dart JK (1995). The management of corneal perforations associated with rheumatoid arthritis. An analysis of 32 eyes Ophthalmology.

[B175] Fogle JA, Kenyon KR, Foster CS (1980). Tissue adhesive arrests stromal melting in the human cornea. Am J Ophthalmol.

[B176] Moorthy S, Jhanji V, Constantinou M, Beltz J, Graue-Hernandez EO, Vajpayee RB (2010). Clinical experience with N-butyl cyanoacrylate tissue adhesive in corneal perforations secondary to herpetic keratitis. Cornea.

[B177] Bodaghi B, Lévy C, Votan P, Hoang-Xuan T (1996). [Value of cyanoacrylate tissue adhesives in peripheral corneal ulcers of inflammatory origin]. J Fr Ophtalmol.

[B178] Sharma N, Singhal D, Maharana PK, Vajpayee RB (2019). Tuck-in tenon patch graft in corneal perforation. Cornea.

[B179] Erikitola O-oC, Jawaheer L, Ramaesh K, Anijeet D (2016). Conjunctival resection for peripheral ulcerative keratitis (PUK). Invest Ophthalmol Vis Sci.

[B180] Raizman MB, Sainz de la Maza M, Foster CS (1991). Tectonic keratoplasty for peripheral ulcerative keratitis. Cornea.

[B181] Speaker MG, Arentsen JJ, Laibson PR (1989). Long-term survival of large diameter penetrating keratoplasties for keratoconus and pellucid marginal degeneration. Acta Ophthalmol Suppl.

[B182] Lohchab M, Prakash G, Arora T, Maharana P, Jhanji V, Sharma N, et al (2019). Surgical management of peripheral corneal thinning disorders. Surv Ophthalmol.

[B183] Maeno A, Naor J, Lee HM, Hunter WS, Rootman DS (2000). Three decades of corneal transplantation: indications and patient characteristics. Cornea.

[B184] Jhanji V, Young AL, Mehta JS, Sharma N, Agarwal T, Vajpayee RB (2011). Management of corneal perforation. Surv Ophthalmol.

[B185] Bessant DA, Dart JK (1994). Lamellar keratoplasty in the management of inflammatory corneal ulceration and perforation. Eye.

[B186] Liu J, Shi W, Li S, Gao H, Wang T (2015). Modified lamellar keratoplasty and immunosuppressive therapy guided by in vivo confocal microscopy for perforated Mooren's ulcer. Br J Ophthalmol.

[B187] Artaechevarria Artieda J, Estébanez-Corrales N, Sánchez-Pernaute O, Alejandre-Alba N (2020). Peripheral ulcerative keratitis in a patient with bilateral scleritis: medical and surgical management. Case Rep Ophthalmol.

[B188] Wassmer C-H, Berishvili E (2020). Immunomodulatory properties of amniotic membrane derivatives and their potential in regenerative medicine. Curr Diab Rep.

[B189] Liu T, Zhai H, Xu Y, Dong Y, Sun Y, Zang X, et al (2012). Amniotic membrane traps and induces apoptosis of inflammatory cells in ocular surface chemical burn. Mol Vis.

[B190] Hanada K, Shimazaki J, Shimmura S, Tsubota K (2001). Multilayered amniotic membrane transplantation for severe ulceration of the cornea and sclera. Am J Ophthalmol.

[B191] Solomon A, Meller D, Prabhasawat P, John T, Espana EM, Steuhl K-P, et al (2002). Amniotic membrane grafts for nontraumatic corneal perforations, descemetoceles, and deep ulcers. Ophthalmology.

[B192] Ngan ND, Chau HT (2011). Amniotic membrane transplantation for Mooren's ulcer. Clin Exp Ophthalmol.

[B193] Lambiase A, Sacchetti M, Sgrulletta R, Coassin M, Bonini S (2005). Amniotic membrane transplantation associated with conjunctival peritomy in the management of Mooren's ulcer: a case report. Eur J Ophthalmol.

[B194] Ke L, Shen D, Wang H, Qiao C, Zeng Q (2020). Lamellar keratoplasty combined with amniotic membrane transplantation for the treatment of corneal perforations: a clinical and in vivo confocal microscopy study. Biomed Res Int.

[B195] Jerez-Peña M, Salvador-Culla B, de la Paz MF, Barraquer RI

